# Soil Food Web Changes during Spontaneous Succession at Post Mining Sites: A Possible Ecosystem Engineering Effect on Food Web Organization?

**DOI:** 10.1371/journal.pone.0079694

**Published:** 2013-11-19

**Authors:** Jan Frouz, Elisa Thébault, Václav Pižl, Sina Adl, Tomáš Cajthaml, Petr Baldrián, Ladislav Háněl, Josef Starý, Karel Tajovský, Jan Materna, Alena Nováková, Peter C. de Ruiter

**Affiliations:** 1 Institute of Soil Biology, Biology Centre Academy of Sciences of the Czech Cepublic, České Budějovice, Czech Republic; 2 Institute for Environmental Studies, Charles University, Prague, Czech Republic; 3 Laboratoire Biogéochimie et Ecologie des Milieux Continentaux Unité mixte de recherche 7618, Centre National de la Recherche Scientifique, Paris, France; 4 Department of Soil Science, University of Saskatchewan, Saskatoon, Canada; 5 Institute of Microbiology Academy of Sciences of the Czech Cepublic, Prague , Czech Republic; 6 Krkonoše National Park, Museum of Krkonoše in Vrchlabí, Vrchlabí, Czech Republic; 7 Biometris, Wageningen University, Wageningen, The Netherlands; Northwestern University, United States of America

## Abstract

Parameters characterizing the structure of the decomposer food web, biomass of the soil microflora (bacteria and fungi) and soil micro-, meso- and macrofauna were studied at 14 non-reclaimed 1– 41-year-old post-mining sites near the town of Sokolov (Czech Republic). These observations on the decomposer food webs were compared with knowledge of vegetation and soil microstructure development from previous studies. The amount of carbon entering the food web increased with succession age in a similar way as the total amount of C in food web biomass and the number of functional groups in the food web. Connectance did not show any significant changes with succession age, however. In early stages of the succession, the bacterial channel dominated the food web. Later on, in shrub-dominated stands, the fungal channel took over. Even later, in the forest stage, the bacterial channel prevailed again. The best predictor of fungal bacterial ratio is thickness of fermentation layer. We argue that these changes correspond with changes in topsoil microstructure driven by a combination of plant organic matter input and engineering effects of earthworms. In early stages, soil is alkaline, and a discontinuous litter layer on the soil surface promotes bacterial biomass growth, so the bacterial food web channel can dominate. Litter accumulation on the soil surface supports the development of the fungal channel. In older stages, earthworms arrive, mix litter into the mineral soil and form an organo-mineral topsoil, which is beneficial for bacteria and enhances the bacterial food web channel.

## Introduction

Mechanisms behind ecological succession have been a lasting topic in ecosystem studies [1], [2]. It has frequently been emphasized that interactions among plants and between plants and their environments drive succession [2]–[4]. Most studies on this issue, however, have considered only vegetation patterns, ignoring community assembly processes at other trophic levels than primary producers [5]. Here we focus on the development of the decomposer food web during succession. The decomposer food web substantially affects the nutrient status of soil [6], [7], and changes in the decomposer food web may have an effect on plant succession [8], [9].

The decomposer food web processes a majority of organic matter produced in terrestrial ecosystems [10]. Fungi and bacteria play a principal role in primary decomposition of dead organic matter, while the direct contribution of detritivorous invertebrate consumers in terms their digestion and assimilation is assumed to be low, although they may alter conditions for soil microorganisms and affect the decomposition rate indirectly [10]. Moreover, soil invertebrates feeding on dead organic matter also ingest microorganisms associated with and embedded in the organic matter. Since the nutritional value of micro-organisms is much higher than that of dead plant material, they become crucial food source for other organisms in decomposer food webs [10], [11]. In this context, the bacterial and fungal pathways of the decomposer food web require particular attention in soil food web studies [12]. Proportion of bacterial and fungal pathway in the food web has significant consequences for ecosystem functioning. The bacterial pathway is generally associated with processing more easy decomposable litter and promotes faster mineralization and nutrient release. On the other hand, prevalence of the fungal pathway is associated with slower mineralization, lower nutrient availability and carbon sequestration [6], [7], [10], [13]. During terrestrial succession in temperate zones, the soil food web is assumed to change from a bacterial to a fungal dominated system [6], [14]. The soil food web is also hypothesized to be bottom-up regulated [15], while succession changes are driven by changes in the plant community with consequent changes in litter quality [6], [16]. However, the soil biota also substantially modifies its environment by bioturbation, aggregate formation, aeration and soil mixing [7], [10]. Some soil invertebrates, such as earthworms, may have strong engineering effects on soil and may completely alter humus forms, causing dramatic shifts in soil properties [7], [10]. The consequences of these engineering effects on soil food web structure are still largely unknown.

In order to better understand primary succession of soils, we studied the soil food webs in a mine spoil chronosequence. We hypothesized that (1) because the amount of organic matter increases during succession, the soil supports more trophic levels and more complex food webs (higher connectance and diversity) following Odum's theory of ecosystem succession [4], [5]; (2) as succession proceeds, the food web changes from a bacterial channel dominated food web towards a more fungal channel dominated one [14]; (3) thickness of fermentation (Oe) layer would be a good predictor of fungal to bacterial channel. Thickness of fermentation layer is driven by several processes, including litter input and bioturbation. Input of litter, namely litter with high CN ratio such as birch, alder or willow litter increase Oe layer thickness on the other hand bioturbation by soil fauna namely earthworms may decrease it [9].

We sampled fourteen decomposer food webs along a chronosequence of post mining sites ranging in age from 1 to 41 years [9], [17] and compared these food web structures with previous studies of soil organic layer development. Post-mining sites are very suitable for this type of study because soil develops here anew from the parent material. Moreover, previous studies show that the presence of earthworms at these sites causes massive changes in soil morphology and plant community composition [9], [17], [18].

## Materials and Methods

### Ethics Statement

Land, where study was performed, has been owned by mining company “Sokolovská Uhelná a.s. právní nástupce”, which permitted access to the sites as well as all filed works done in this study and also provided data about plot age. As concern other rules and regulations no permits were required for the described study, which complied with all relevant regulations.

### Study site

The study was carried near the town of Sokolov in the western part of the Czech Republic, Central Europe on four large colliery spoil heaps (from several dozen to several hundred km^2^ in area) resulting from open-cast coal mining. The altitude of the study area was 500–600 m a.s.l. with mean annual precipitations of 650 mm and mean annual temperature of 6.8°C. A chronosequence of 14 non-reclaimed sites, created 1–41 years ago using the same substrate, was available for the study [9], [19]. At all of the sites selected for the study, no subsequent manipulation of the heap material occurred, and the sites have developed on their own since the heaping. At each site, a sampling plot (10×10 m) was established at least 5 m from the site margin. Plot age (time since heaping) was determined based on historical data supplied by the coal mining company. The substrate of all the sites was composed of alkaline tertiary clay shales, substrate do not show any sign of contamination, site pH gradually decreased with plot age, as plant succession progressed (Table 1). As a result of the heaping process, parallel rows of depressions and elevations were formed at each site. Because of remarkable changes in vegetation soil development, we called these succession stages the initial, the shrub and the forest stage; the initial stage covered four sites 1–14 years old, the shrub stage six sites 15–22 years old and the forest stage four sites 23–41 years old [9], [19]. In the initial stage (i.e., 1–14 years before the establishment of the shrub layer), the vegetation was dominated by a sparse herb cover, and the topsoil formed mainly from the dumped spoil material. At the 14–22 year-old sites dominated by shrubs (namely *Salix caprea*), the litter reserve on the soil surface was higher than annual litter production, and a thick fermentation layer developed. In older plots (24 years old or older), dominated by a young birch and aspen (*Betula pendula* and *Populus tremula*) forest, the humus layer was formed, and the thickness of the fermentation layer decreased (Table 1).

**Table 1:** Characteristics of soil development and plant production in individual succession stages.

	pH		Oe		A		herb mass		litter input		fine roots	
			cm		cm		g m^−2^		g m^−2^ year^−1^		g m^−2^	
initial	7.9	±0.4	0.1	±0.1	0.0	±0.0	42	±34	8	±14	63	±78
shrub	7.2	±0.3	2.4	±0.1	0.0	±0.0	22	±35	152	±87	594	±3
forest	6.6	±0.3	0.6	±0.2	3.4	±1.7	111	±76	268	±97	463	±194

Values represent mean ± SD for individual phases of succession obtained by pooling data from 4 initial, 6 shrub and 4 forest sites, the initial, shrub and forest stages were 1–13, 14–22 and 23–41 years old, respectively. Oe refers to fermentation layer thickness and A to the thicknes of the darker organo-mineral layer. Based on data in Frouz et al. [9].

### Sampling and analysis

Soil microflora sampling and processing is described in Frouz and Nováková [19]. Composite soil samples consisting of 5 individual subsamples (each consisting of about 100 g of soil) were sampled in March 2002 from top 5 cm below the litter layer (see Frouz and Nováková [19] for more details). Both microhabitat depressions and wave tops were sampled separately, but pooled data are presented here. Microbial biomass was measured by the chloroform fumigation and extraction method [20]. Biomass was assumed to be twice the mass of C. The amount of fungal biomass was estimated by measuring ergosterol content (see Baldrian et al. [21] for more details).

The soil fauna was sampled in March 2002 as described in Frouz et al. [9]. To study the soil microfauna and mesofauna, two composite samples (one from depressions and the other from elevations) were taken from each plot, each consisting of five individual samples (area of each 10 cm^2^, depth 5 cm). Two sets of samples were collected, one for the extraction of microfauna and enchytraeids and the other for the rest of the mesofauna. Samples of the first set were homogenized; 1 g of the mixed soil was diluted and used to directly count protists following standard methods [22]. Additional 10 g of the soil were exposed in Baermann funnels modified according to Háněl [23] to isolate the metazoan microfauna (nematodes, rotifers and tardigrades) and enchytraeids. A Tullgren apparatus was used for the extraction of other mesofauna groups.

To study the soil macrofauna, two composite samples (one from depressions and the other from elevations), each consisting of five particular samples (area of each 125 cm^2^, depth 5 cm), were taken in each plot. The macrofauna were extracted by a Kempson apparatus.

**Figure 1:**
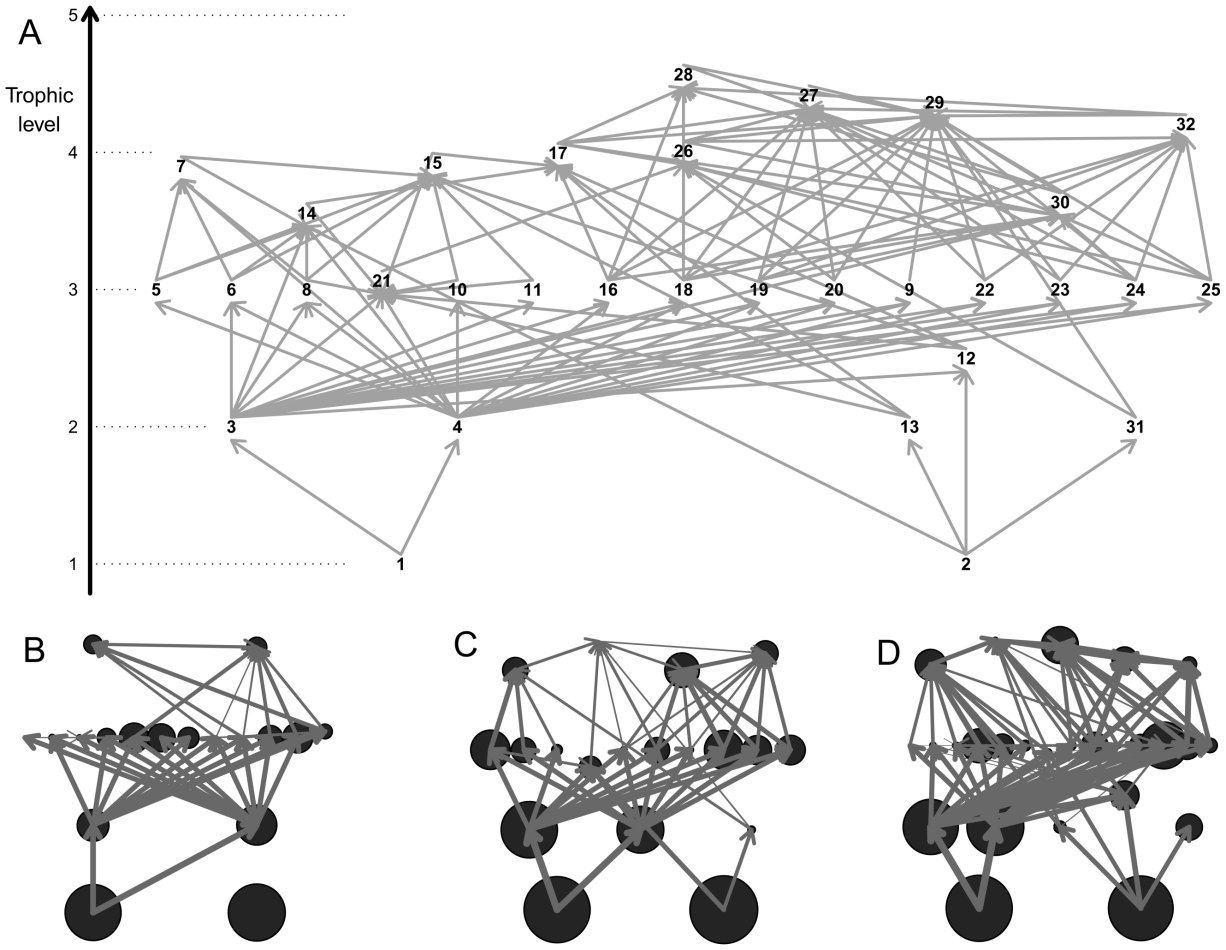
Food web diagrams in the post mining chronosequence. **A**. General food web diagram as defined in this study. **B**, **C** and **D**. Examples of soil food webs observed in the initial (**B**), shrub (**C**) and forest (**D**) stages. In panel **A**, the numbers refer to the trophic groups: 1, detritus; 2, roots; 3, fungi; 4, bacteria; 5, naked amoebae; 6, flagellates; 7, ciliates; 8, testacea; 9, enchytraeidae; 10, bacteriophagous nematodes; 11, fungivorous nematodes; 12, root and fungal feeder nematodes; 13, plant parasite nematodes; 14, omnivorous nematodes; 15, predatory nematodes; 16, oribatida; 17, predaceous acari; 18, collembolan; 19, other microarthropods; 20, litter dwelling lumbricidae; 21, mineral dwelling lumbricidae; 22, isopoda; 23, diplopoda; 24, microsaprophagous diptera; 25, macrosaprophagous diptera; 26, predaceous diptera; 27, chilopoda; 28, aranea; 29, carabidae; 30 staphylinidae; 31, root feeding colleoptera and 32, formicidae. The arrow on the left indicates the trophic level of the groups, with primary producers and detritus at the bottom of the food web (trophic level  = 1). In panels **B**, **C** and **D**, the size of the circles correspond to the relative biomass of the different trophic groups in the food web, while the width of the arrows corresponds to the relative intensity of C flow between trophic groups estimated by the model.

In total 30 guilds of soil biota was distinguished, two guilds of soil microflora (bacteria and fungi), four guilds of Protozoa (naked. amoebae, flagellates, ciliates and testacea) and 24 guilds of soil fauna. The soil fauna were sorted into 24 guilds according to the following ad hoc guild system. Taxonomic groups of the soil fauna with rather uniform ecology were considered as guilds. Other taxonomic groups were divided into several guilds based on their feeding habits. Nematodes (Nematoda) were divided into bacterivores (Nem-Bact), fungivores (Nem-F), root and fungal feeders (Nem-RFF), plant parasites (Nem-PP), predators (Nem-P), and omnivors (Nem-O) [24]. Plant-associated nematodes are herein referred to either as root fungal feeders - consisting from members of family Tylenchidae that feed on plant roots but also on fungi, and plant parasites that feed on various parts of plants and cause plans diseases [25]. Diptera larvae were divided into microsaprophags, saprophags and predators according to Frouz [26]. Earthworms (Lumbricidae) were divided into two groups: litter dwelling epigeic earthworms and mineral soil dwellers. The first group includes species such as *Dendrodrilus rubidus* and *Dendrobaena octaedra*, which live preferentially in the litter layer. The latter includes endogeic species and the epigeic species *Lumbricus rubellus*, which lives in litter as well as at the top of the mineral layer [27], [28]. This ad hoc system for earthworms was used because anectic species were completely absent from the investigated plots and endogeic species were rare. The biomass of each group was estimated based on allometric equations. Carbon was estimated based on the assumption that 50% of biomass is carbon. Characteristics of individual feeding guilds were used to produce a topographic stucture of food web (Figure 1).

### Estimation of feeding rates in the soil food web

In order to estimate annual feeding rates from the observed trophic group biomasses, we followed Hunt et al. [29] by assuming that species production balances out losses through natural death and predation (assumption of steady state). Trophic group feeding rates can thus be expressed as:
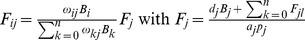
(4)where *F_ij_* is group *j* feeding rate on group *i* (g m^−2^ yr^−1^) which depends on *F_j_*, the group *j* overall feeding rate (g m^−2^ yr^−1^) and on the relative biomass *B_i_* of group *i* and feeding preference *ω_ij_* of *j* on *i*. The feeding preferences, death rates, assimilation and production efficiencies of the different trophic groups were taken from Hunt et al. [29] and further updated by a review of recent literature on the subject (see Table 2).

**Table 2:** Trophic groups.

Trophic group	Initial		Shrub		Forest		A	P	D
Fungi	4106	±4659a	43853	±28161b	31354	±12629ab	1	0.44	3.7
Bacteria	6404	±6791a	6787	±3603a	47031	±18944b	1	0.51	9
Nak. Amoebae^1^	+		+		+		0.55	0.58	7.3
Flagellates	+		+		+		0.52	0.6	7.3
Ciliates	+		+		+		0.55	0.58	7.3
*Testacea*	34	±29a	715	±219b	223	±51ab	0.55	0.58	7.3
Enchytraeidae	22	±39a	8	±18a	267	±164b	0.28	0.29	1.95
*Nem-Bact^2^*	89	±112	72	±12	14	±7	0.54	0.49	14.1
Nem-F^3^	4	±5	1	±1	1	±0	0.42	0.31	6
Nem-RFF^4^	1	±1	+		3	±3	0.42	0.31	6
Nem-PP^5^	+		1	±6	5	±6	0.42	0.31	2.3
Nem-O^6^	146	±211	164	±120	77	±49	0.55	0.28	5.8
Nem-P^7^	13	±17	44	±57	54	±49	0.55	0.28	5.8
*Oribatida*	+		+		+		0.5	0.4	1.42
Pred. acari^8^	+		+		+		0.75	0.3	3.44
*Collembola*	+	a	24	±25b	47	±25b	0.34	0.37	1.96
Other microart^9^			+		+		0.34	0.37	1.96
Lumb liter^10^			+		65	±101	0.22	0.32	0.14
Lumb min^11^		a	++	1a	3040	±2357b	0.22	0.32	0.14
Isopoda			1	±390	9	±16	0.25	0.18	1
Diplopoda	155	±252	5	±119	254	±407	0.25	0.18	0.5
Dip. micro^12^	46	±40			27	±20	0.50	0.17	3.33
Dip. sap^13^	20	±35a	+	a	400	±265b	0.35	0.125	1
Dip. pred^14^	84	±145	40	±20	12	±21	0.60	0.17	1
Chilopoda		a	284	±195b	546	±125c	0.34	0.20	1
Aranea	15	±13ab	2	±32a	33	±18b	0.72	0.18	1
Carabidae	10	±13	41	±195	13	±13	0.80	0.17	1
Staphylinidae	4	±3	9	±*	1	±1	0.50	0.17	1
Root f. Col^15^	7	±12a		a	34	±26b	0.50	0.17	0.5
Formicidae			19	±19	5	±4	0.30	0.10	1
									
***Sum***	11161	±12024a	52584	±29479a	83517	±33404b			

Mean and SD of biomass dry weight (mg m^−2^) in individual sucession stages and values of physiological parameters of the different trophic groups in the food web. A – assimilation efficiency, P – production efficiency, D – death rate (yr^−1^), data from Hunt et al. [29]. Biomass values lower than 0.5 mg are marked as +; empty cells indicate the absence of a group. Statistically homogeneous groups of the same guild in various succession stages are marked by the same letter (ANOVA, LSD test p<0.05); no letter means that no significant difference was found.

1 – Naked Amoebae, 2 – Nematodes, bacteriophagous, 3 – Nematodes, fungivorous, 4 – Nematodes, root and fungal feeders, 5 – Nematodes, plant parasites, 6 – Nematodes, omnivores, 7 – Nematodes, predators, 8 – Predaceous acari, 9 – Other microarthropods, 10 –Lumbricidae, litter dwelling, 11 – Lumbricidae, mineral dwelling, 12 – Diptera, microsaprophagous, 13 – Diptera, macrosaprophagous, 14 –Diptera, predaceous, 15 – Colleoptera, root feeding.

The estimation of the feeding rates requires solving a system of equations (4) with all trophic groups. In a few instances (4 out of 14 food webs), it was not possible to solve this system because of intraguild predation between two top predators (Aranea and Formicidae). In this case, we consider that only the most abundant predator consumes the other, and thus we ignore one trophic link when estimating feeding rates.

### Analyses of soil food web structure

We analysed several parameters of the food web structure. First, food web richness (*S*) corresponds to the number of groups in the food web, while food web diversity (*DivS*) is calculated as 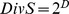 with 
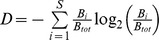
 and *B_i_* is the biomass of trophic group *i* and *B_tot_* the total food web biomass (Shannon diversity index). Food web connectance is expressed as the number of realized links in the food web over the number of possible links.

Secondly, we also consider measures based on trophic position and measures based on energy channels. The trophic position of a species is defined here by the average of the trophic position of the species it consumes weighted by the diet fraction these species represent:


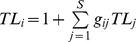
 Where TL_i_ is the trophic level of species *i* and *gij* the fraction of the consumer *i*'s diet derived from the prey *j*. These “flow-based” trophic levels were computed following the method of Levine (1980) [30]. The average trophic level for each consumer is the sum of all entries in each column of *A* =  [*I* –*G*]^−1^ with *I* being the identity matrix and *G* =  (*gij*). The food web structure is characterized here by the average trophic level (TLm), calculated as average of all values of group trophic levels in the food web and by the maximum trophic level (TLM), corresponding to the maximum group trophic level in the food web.

Fungal and bacterial energy channels are measured by the biomass of all the groups belonging to that channel weighted by their contribution to this channel. The contribution of a group to a channel is defined by 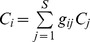 and thus the contribution of each group is equal to the product of *A* by a vector *V*, with *V_i_* = 1 for the source of the energy channel (either fungi or bacteria) and 0 otherwise. We measured two different indices to quantify the fungal and bacterial energy channel. First we summed the biomass of all the groups belonging to a given channel weighted by their contribution *C* to this channel. Then, because the order of magnitude of biomasses differs strongly between the trophic groups, we also calculated the energy channels with standardized biomasses of each group by dividing the biomass of one group by the overall mean of that group over all food webs [31].

We estimated the stability of the soil food webs by following the method of de Ruiter et al. [32] and Neutel et al. [5], [33]. Stability is evaluated on the Jacobian community matrix, following May's approach [34]. We estimate the Jacobian matrix corresponding to each food web as a function of the trophic group biomasses and feeding rates calculated above and by assuming that species dynamics can be modelled by using the generalized Lotka-Volterra system near the equilibrium [33]. Food web instability is defined as the level of intraspecific interaction (diagonal strength) needed for all eigenvalues of the Jacobian matrix to have negative real parts. Food webs that need high levels of diagonal strength are more instable.

A one way ANOVA was used to compare individual food web parameters among individual succession stages. Trends between individual parameters and plot age were fitted by linear, logarithmic, exponential and or second order polynom functions. We present function that was significant and explain the largest proportion of data variability.

## Results

**Figure 2:**
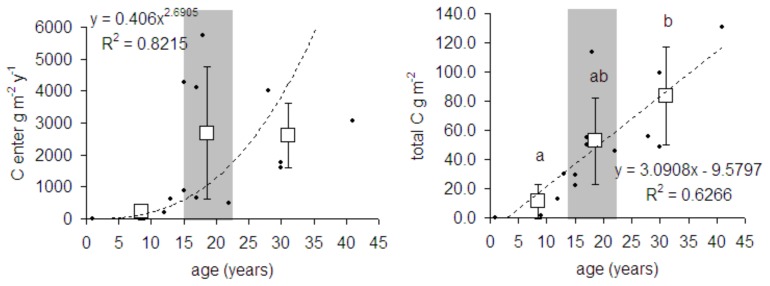
Food web C flow and total biomass of individual food webs related to plot age. Amount of C entering food web ner m^2^ and year, and biomas per m^2^. Dots indicate the parameter value for individual ages; squares correspond to the mean value (bars mean SD) for initial, shrub and forest stages. Grey areas highlight the shrub stage and delimitate the initial stage on the left and the forest stage on the right. A trend line between a given parameter and time is present only if significant p<0.05; statistically homogeneous groups of stages are indicated by the same letter (ANOVA, LSD post hoc test p<0.05); if no letter is present in the panel, no significant difference was found.

**Figure 3:**
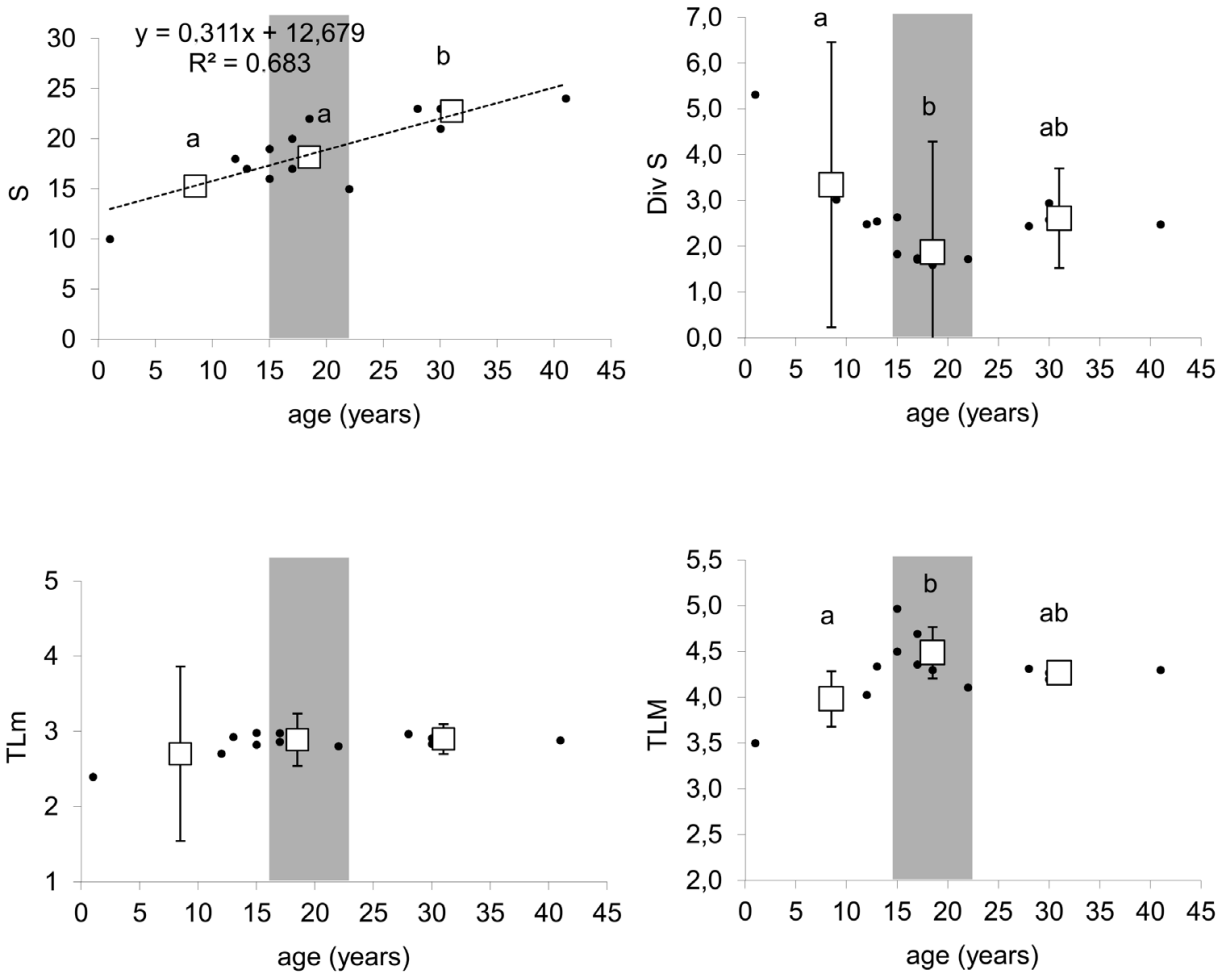
Food web diversity related to plot age. Number of functional groups in the food web (S), exponent of Shannon-Weiner index, of diversity for functional groups present in the food web (Div S), mean (TLm) and maximum (TLM) trophic level of the groups in the food web. Dots indicate the parameter value for individual ages; squares correspond to the mean value (bars mean SD) for initial, shrub and forest stages. Grey areas highlight the shrub stage and delimitate the initial stage on the left and the forest stage on the right. A trend line between a given parameter and time is present only if significant p<0.05; statistically homogeneous groups of stages are indicated by the same letter (ANOVA, LSD post hoc test p<0.05); if no letter is present in the panel, no significant difference was found.

**Figure 4:**
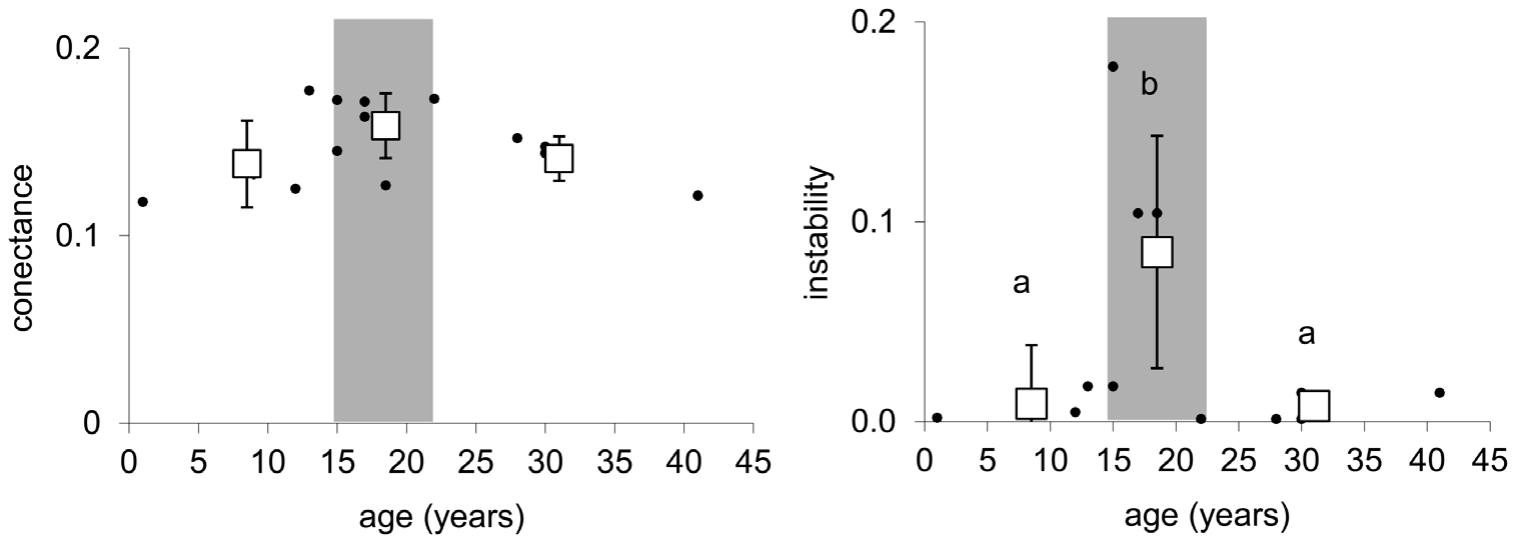
Conectance and instability of food webs related to plot age. Dots indicate the parameter values for individual ages; squares correspond to the mark mean value (bars mean SD) for initial, shrub amd forest stages. Grey areas highlight the shrub stage and delimitate the herb stage on the left and the forest stage on the right. There is no significant trend line between the given parameters and time. Statistically homogeneous groups of stages are indicated by the same letter (ANOVA, LSD post hoc testp<0.05).

During succession, the biomass of the whole food web, as well as the amount of C that flows through it increased (Figure 2, Table 2). The number of functional groups was significantly positively correlated with plot age (Figure 3). Also the maximum trophic level of the groups in the food web, which indicates the length of the food chain in the food web, increased with succession age. However, this increase was the most pronounced during the first years of succession before shrub vegetation developed. Later on, the maximum trophic level of the groups in the food web did not differ significantly between the shrub and the initial stage (Figure 3). The mean trophic level of the groups did not differ significantly between succession stages (Figure 3). We did not detect any significant changes in food web connectance during succession (Figure 4). Food web instability in the shrub stage was significantly higher than in the other stages (Figure 4).

**Figure 5:**
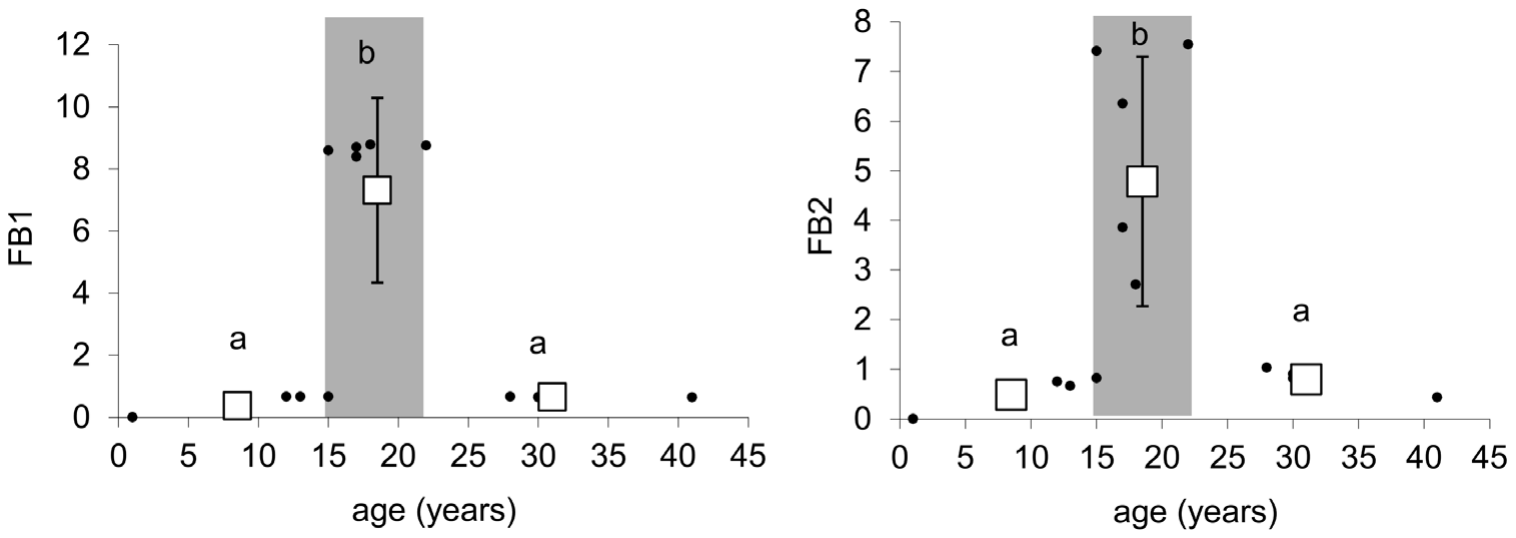
Proportion of the fungal to the bacterial channel. FB1 -based on the biomass of individual guilds, FB2 based on the biomass of individual guilds standardized over the biomass of the same guild in the whole chronosequence. Dots indicate the parameter values for individual ages; squares correspond to the mark mean value (bars mean SD) for initial, shrub and forest stages. Grey areas highlight the shrub stage and delimitate the herb stage on the left and the forest stage on the right. There is no significant trend line between the given parameters and time. Statistically homogeneous groups of stages are indicated by the same letter (ANOVA, LSD post hoc test p<0.05).

**Figure 6:**
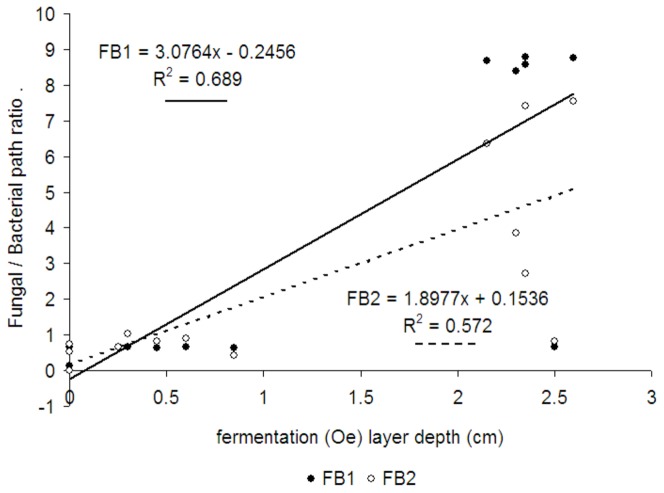
Relationship between fungal bacterial ration and Oe layer. Relationships between thiskness of fermentation Oe layer and two forms of fungal bacterial pathway ratio, FB1 -based on the biomass of individual guilds, FB2 based on the biomass of individual guilds standardized over the biomass of the same guild in the whole chronosequence. Both regression lanes are significant p<0.05.

Initial food webs were dominated by the bacterial channel, but in the shrub stage and intermediate stages of succession, there was a strong and significant increase in the ratio of the fungal over the bacterial channel, and the intermediate stages of succession were strongly dominated by fungi (Figure 5, Table 2). Later on in forest stages, the proportion of the fungal over the bacterial channel decreased significantly. In both the initial and the forest stage, the food webs were therefore dominated by the bacterial channel (Figure 5). Considering the environmental variables in Table 1, thickness of fermentation layer (Oe horizon) was the best predictor of the fungal over the bacterial channel (Figure 6). Other parameters in Table 1 did not show any significant correlation with the fungal/bacterial channel ratio.

## Discussion

Remarkable shifts from the bacterial to the fungal channel and back were observed during the succession. The increase of the fungal channel compared to the initial bacteria-dominated channel in the shrub stage is consistent with the general assumption that the importance of the fungal channel should grow during succession [6]. On the contrary, the decrease in the importance of the fungal channel and the prevalence of the bacterial channel observed in the next forest stage seems to be against this expectation. As shown above, the thickness of the fermentation (Oe) layer is the best predictor of the fungal/bacterial channel ratio. The decrease in the fungal bacterial channel ratio at the transition from the shrub to the forest stage corresponds with a decrease in the thickness of the fermentation layer. Previous micromorphological observations [9], [17], [19] on the same chronosequence show that this decrease is associated with colonization of these sites by earthworms, which take part in bioturbation and mixing of litter and the fermentation layer in mineral soil. This mixing is also associated with changes in the soil microbial community, namely an increase in populations of bacteria and a decrease in the fungal/bacterial ratio [18], [19], [21] and may explain the observed changes in the fungal to bacterial channel ratio. The observed decrease in the importance of the fungal over the bacterial channel and the dominance of the bacterial channel in forest stages of succession might have therefore been caused by engineering effects of earthworms, which remove the fermentation layer that is favourable for fungi by bioturbation. In addition, consumption, digestion and excretion of the fermentation layer also promotes the growth of bacteria. This is consistent with previous studies describing the effects of earthworm invasion on soil and the microbial community in particular [19], [35]. In the present study, the removal of the litter accumulation and fermentation (Oe) layer from the soil surface by earthworm bioturbation is assumed to be the major factor promoting bacteria over fungi [36]. Changes of humus forms from mor-moder to moder-mull associated with earthworm colonization were observed not only in this chronosequence but also on other chronosequences of primary succession [37]–[40]. Consequent changes in the microbial community are in agreement with the concept of humus form where moder-like forms of humus with little bioturbation are dominated by fungi whereas mull-like forms with intensive bioturbation are dominated by bacteria [7], [10]. The effect of earthworms bioturbation on the soil community is complex and consists of effects of worm on vegetation and soil [9], [41]. Similar mechanisms may apply also in situations when earthworm free ecosystems are invaded by earthworms, such as north temperate forests in USA [35].

In our opinion, this observation cannot be interpreted that this particular chronosequence develops into a bacteria-driven food web at the climax stage. Instead, we presume that the proportion between the fungal and the bacterial channel may change several times over the course of succession as soil organic matter accumulates and the soil community matures from a functional ecology perspective as well as from a biodiversity perspective. The oldest observed stages in the present study are now dominated by an aspen and birch forest, but there are already numerous beech and oak seedlings in the understory [9]. A beech and oak forest represents the hypothetical (historical) climax stage in this region. Further development toward a beech and oak dominated climax forests may bring about further changes in litter quality and cause another shift to a fungal food web. We rather tend to interpret the observed shifts in food web channels as intermediate succession changes.

One interesting question is how much can this pattern of successional development be related to soil development in general and how much is the observed successional pattern a mere exception in early soil genesis and primary succession situations. Similar waves in plant diversity are often observed during plant community succession [42]–[44]. Adl [45] also pointed out that changes in the soil food web and community structure occur after about 20 years of succession, which is likely related to soil structure and horizon development. Kaufmann [46] observed based on comparison of long-term data and chronosequence data that community development at individual sites often does not follow idealized succession trajectories that can be derived from chronosequences, which, again, suggests that successional trajectories are likely to consist of one or several oscillating waves rather than a gradual transition from one stage to another.

The effect of ecosystem engineers on food web structure is frequently discussed [47], [48] but seldom observed. One of the reasons for this is that the effect is indirectly related to environmental changes mediated by particular ecosystem engineers. These changes may be slow, so it may take some time after engineering species occur before changes in the ecosystem can be detected and, vice versa, existing structures may persist for some time after engineering species are removed.

The observed increase in overall food web biomass and diversity with increasing succession age is in good agreement with at least one previous study [14]. As concern development of individual taxa oribatids, which are bioindicators of site disturbance, it is likely that the sites are still too immature to provide an adequate habitat. Others such as predatory consumers (nematodes, acarids) appear when there is sufficient prey in the food web, that is, in later succession stages. Similarly, collembola and root feeding nematodes appear later when there is sufficient root biomass. The biomass of protists is always low in soil; their contribution to biomass turnover (bacteriophagy) is nonetheless significant [49]–[51].

We did not detect changes in food web connectance even though other studies found positive results [5]. Food web instability in shrub dominated intermediate stages of succession increased, so stability decreased. This is consistent with previous observations of Neutel et al. [5] who suggest an alternating pattern of decreasing and increasing stability in the course of ecosystem succession. According to Neutel et al. [5], this stability fluctuation is connected with biomass accumulation at the top trophic level, and a decrease in stability at the point of creation of a new food web structure is caused by the arrival of a new top predator. In our case, the changes in food web structure included not only additional new trophic levels but also shifts in flows between the fungal and the bacterial pathway (as described above), which may contribute to the alteration of food web stability. A recent theoretical study indeed suggests that coupling of two different energy pathways in food webs can strongly determine ecosystem stability [52]. It would be difficult to generalize the outcome of our result based on one chronosequence, but exploring the role of pathway proportion in food web stability clearly represents a promising field for future research.
